# Evaluation of a treatment algorithm for acute traumatic osseous Bankart lesions resulting from first time dislocation of the shoulder with a two year follow-up

**DOI:** 10.1186/1471-2474-14-305

**Published:** 2013-10-25

**Authors:** Ulrich J A Spiegl, Christian Ryf, Pierre Hepp, Paavo Rillmann

**Affiliations:** 1 Department of Surgery, Davos Hospital, Promenade 4, Davos Platz, 7270, Switzerland; 2 Department of Trauma and Reconstructive Surgery, University of Leipzig, Liebigstr 20, Leipzig, 04103, Germany

**Keywords:** Glenoid rim fracture, Osseous Bankart lesion, Anterior shoulder dislocation, Operative treatment strategy, Conservative treatment

## Abstract

**Background:**

Studies dealing with acute osseous Bankart lesions and corresponding treatment strategies are rare. The purpose of this study is to analyze the results after applying our treatment algorithm for acute glenoid rim fractures caused by first time traumatic anterior shoulder dislocations.

**Methods:**

25 patients were included in this retrospective case series. All patients sustained a first time shoulder dislocation caused by ski or snowboard accidents. An osseous Bankart lesion was detected in all shoulders. Operative therapy was performed in patients with osseous defects of 5% or more, otherwise conservative therapy was initiated. Primary study outcome parameter was the Rowe score. Additionally, the outer rotation deficit and operative complications were analysed.

**Results:**

12 patients showed a defect size of less than 5% and were treated conservatively. The average lesion size was 2%. For these patients, the Rowe score was excellent in 58%, good in 25%, and moderate in 17% of patients. Three patients (25%) complained about a feeling of instability. 13 patients had a lesion size of more than 5%, average 15%, and were treated operatively. The Rowe score for this group was excellent in 54%, good in 31%, and moderate results in 15% of patients. One patient (8%) complained about a feeling of instability, without recurrent dislocations. There were no statistically significant differences between both study groups (ROWE score: p = 0.98).

**Conclusions:**

Applying our treatment algorithm for acute osseous Bankart lesions consisting of a conservative strategy for small defect sizes and a surgical approach for medium-sized and large defects leads to encouraging mid-term results and a low rate of recurrent instability in active patients.

## Background

The osseous Bankart lesion is an avulsion of the humeral labral complex with an anterior rim fracture. It may result either from a traumatic glenohumeral dislocation or a direct trauma to the adducted arm [1]. The incidence of anterior glenoid rim fractures has been reported to be up to 22% after first time anterior shoulder dislocation and up to 73% after recurrent dislocations [2-7].

Studies dealing explicitly with acute lesions and their corresponding treatment concepts are rare. For such cases, both Porcellini et al. [1] and Millett et al. [8] reported good results after treating acute osseous Bankart lesions arthroscopically; however, Maquieria et al. [9] concluded that even large, displaced glenoid rim fractures can be successfully treated non-operatively if the glenohumeral joint is concentrically reduced. Furthermore, Salomonsson et al. [10] found, that bony Bankart lesions were associated with good functional outcomes in cases of first time dislocations and conservative therapy. Similarly, Vermeiren et al. [11] described a significant reduced recurrent dislocation rate in cases of fracture associated first time anterior shoulder dislocation.

In contrast, Nakagawa at al. [12] recently reported of severe absorption of bone fragments in the majority of patients after primary traumatic shoulder dislocation. Additionally, Porcellini et al. [13] found less favourable outcomes in patients after arthroscopically treatment of chronic bony bankart lesions compared to acute ones.

Only limited evidence regarding the risk factors for recurrent instability and absorption are available for patients with first-time anterior shoulder dislocations and a bony Bankart lesion. However, a direct relationship between instability and osseous lesion size of the glenoid rim was shown in a biomechanical cadaver study after performing Bankart repair [3]. Bigliani et al. [6] classified bony Bankart lesions in three types depending on the lesion size. In the study by Bigliani et al. [6] an osseous repair was recommended in cases of ununited fragments attached to the separated labrum, whereas in cases of malunited fragments and anterior glenoid defects less than 25% a capsular repair was performed. Anterior glenoid defects exceeding 25% were repaired by bone graft from the coracoid. Sugaya et al. [14] classified glenoid rim lesions in patients with recurrent anterior shoulder instability in small (< 5%), medium (5 to 20%), and large lesions (> 20%) by evaluating the percentual osseous defect area with 3D CT reconstruction. Sugaya et al. [14] suggested arthroscopic or open reconstruction with fixation of the osseous fragment in cases with small and medium sized lesions. Based on this information and under consideration of the positive effect of an acute fracture situation regarding outcomes after conservative therapy, we recommended conservative therapy to patients with small, concentric reduced osseous Bankart lesions (<5%) and a surgical approach in cases with medium or large lesions as well as in cases with unstable small lesions, where concentric reduction could not be obtained.

The aim of this study was to analyze the results applying our treatment algorithm for acute glenoid rim fractures caused by first time traumatic anterior shoulder dislocations in an active population.

## Methods

**Figure 1:**
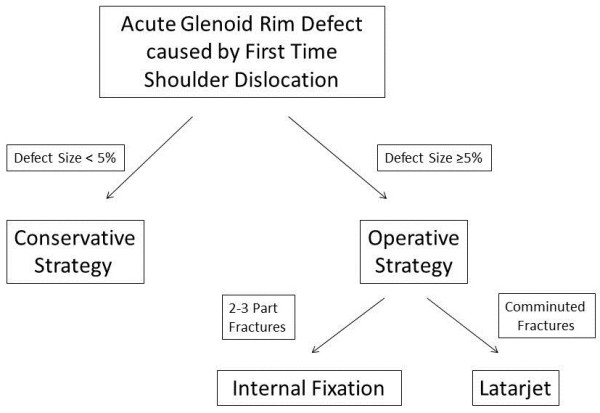
**Flow-chart depicting our treatment algorithm.**

**Figure 2:**
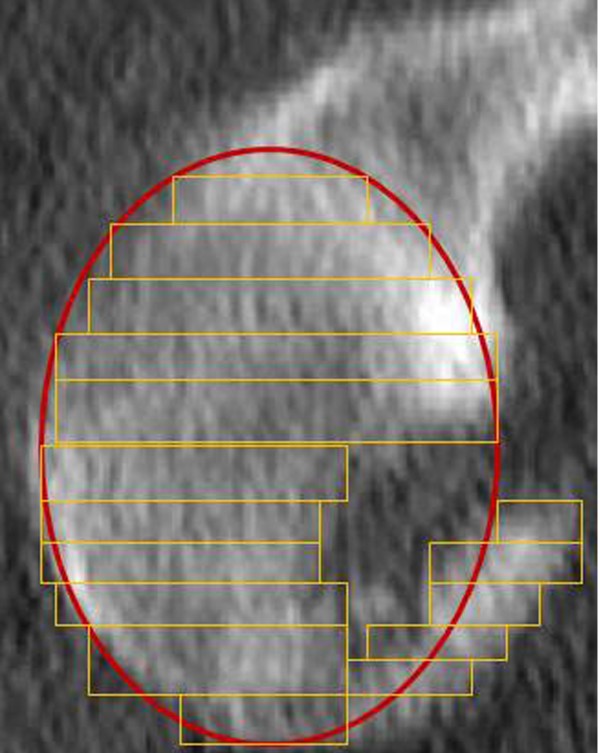
**Sagittal view of a glenoid with an acute osseous bankart lesion.** The outer fitting circle of the glenoid is added. Additionally, multiple rectangles are illustrated, representing the subareas areas of the glenoid size and bony Bankart fracture size. Each rectangle is defined by the width of both, the glenoid and the bankart fragment, multiplied by the slice thickness. The sum of theses rectangles defines the fracture size and the glenoid size.

**Figure 3:**
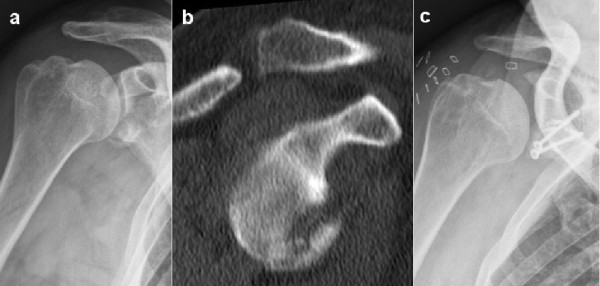
**Case example of a large glenoid rim fracture and a Latarjet procedure.** 60 year old patient with a fresh glenoid rim fracture **(a)** and a defect size of 25% **(b)**. Due to the comminuted fracture situation a Latarjet procedure has been performed **(c)**.

**Figure 4:**
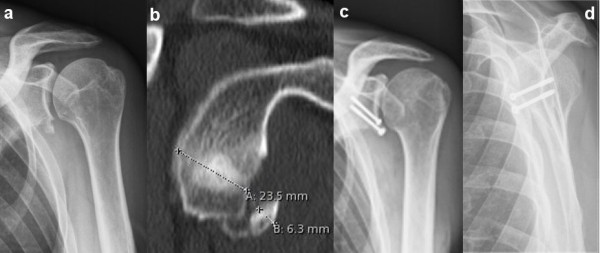
**Case example of a large glenoid rim fracture and screw fixation.** 69 year old female with traumatic anterior shoulder dislocation. **(a)** The conventional x-ray examination showed an osseous bankart lesion with an estimated defect size of 21% **(b)**. Open reduction and internal screw fixation has been performed **(c, d)**.

This is a retrospective case series including all patients who sustained acute traumatic osseous Bankart lesions after first time anterior shoulder dislocation without accompanying rotator cuff tears between November 2004 and May 2006 (Table 1). All patients were active and injured while skiing or snowboarding. Exclusion criteria included prior surgeries of the affected shoulder, neurological deficit after trauma, and a change of the initiated therapy strategy in cases, which were not associated with complications and problems. Of the total 35 patients who met these criteria, twenty-five responded during the follow up period (follow-up rate: 71%). Twenty-one were male (84%), four were female (16%), and the mean age was 50 years (range 22 – 69 years). The dominant arm was affected in 18 cases (72%). There were no professional athletes in our study population. The shoulder dislocation and acute anterior glenoid rim fracture were confirmed by conventional x-ray examination and included standard anteroposterior and transscapular views according to the Neer method. Two patients showed an additional, undislocated tuberculum majus avulsion fracture. All shoulders were reduced in the emergency room. None of the patients had signs of neurologic deficiency after reduction. A CT examination was performed after reduction of the glenohumeral joint in all patients with a glenoid rim fracture on plain radiographs. Recommendation for further treatment depended on fracture size, and fracture configuration according to Figure 1. Fracture size was quantified by CT using 3-D reconstruction to evaluate the percent defect area of the outer fitting circle of the glenoid [14]. The fracture size was quantified by measuring the fragment thickness and glenoid diameters of all axial slices, illustrated in Figure 2. All patients with a fracture size of 5% or more were recommended to undergo operative reconstruction. In all cases reconstruction was performed within the first two weeks after trauma. In four patients (31%) arthroscopic reconstruction was performed using absorbable suture anchors (n = 4), whereas in three patients (23%) an arthroscopic assisted reconstruction was done using absorbable suture anchors with additional small fragment screw osteosynthesis. In six cases (46%) open reconstruction was performed. Two patients were treated by coracoid transfer according to the Latarjet procedure [15,16] (Figure 3). The comminuted fracture situations offered no chance of reconstruction in both cases. The bone loss was 8% and 25%, respectively. In four cases the glenoid rim lesions were reconstructed using small fragment screws (Figure 4) depending on the fracture situation. All operative procedures were performed in beach chair position under general anaesthesia, antibiotic prophylaxis was applied routinely.

**Table 1:** The patient collective

**Age**	**Date of trauma**	**Defect size (%)**	**Therapy**	**Complications**	**Rowe**
63	01 / 06	1	Conservative	None	86
22	02 / 04	1	Conservative	None	100
54	03 / 05	1	Conservative	None	100
52	12 / 05	1	Conservative	None	92
32	02 / 05	1	Conservative	Instability	80
37	01 / 05	1	Conservative	None	100
56	04 / 05	1	Conservative	None	100
54	01 / 06	2	Conservative	None	93
50	01 / 06	2	Conservative	Instability	58
32	04 / 05	2	Conservative	3 Redislocations	51
48	12 / 05	3	Conservative	None	90
47	04 / 04	3	Conservative	None	87
55	03 / 06	8	Latarjet	Arthroscopic release	63
41	02 / 05	8	Suture Anchors (arthroscopic)	None	89
54	02 / 05	11	Screws + Suture Anchors	Instability	71
31	01 / 05	11	Screws + Suture Anchors	None	97
55	03 / 05	11	Screws + Suture Anchors	None	100
57	04 / 05	12	Screws	None	91
41	02 / 06	18	Suture Anchors (arthroscopic)	None	92
64	01 / 05	21	Screws	None	100
69	11 / 05	21	Screws	Arthroscopic release	85
55	02 / 06	21	Suture Anchors (arthroscopic)	None	85
63	12 / 05	25	Screws	None	100
60	03 / 06	25	Latarjet	None	88
55	02 / 06	25	Suture Anchors (arthroscopic)	None	98

**Figure 5:**
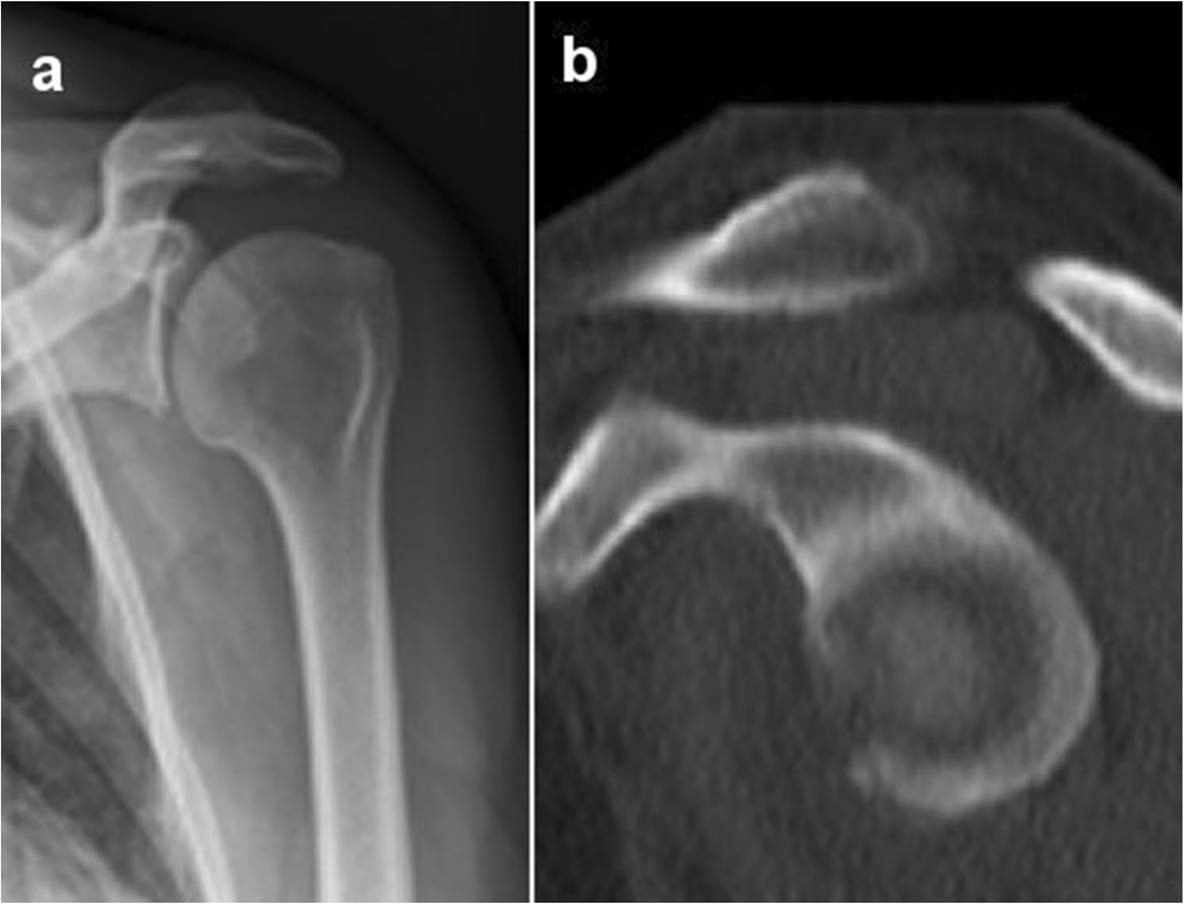
**Case example of a small glenoid rim fracture and conservative management.** 54 year old male who sustained an anterior dislocation of the left shoulder skiing with a small osseous glenoid rim lesion after falling while skiing **(a)**. The defect size was estimated to be 2% **(b)**. The patient was treated conservatively.

**Table 2:** Results

	**n**	**Age**	**Defect size (n)**			**Rowe score (n)**				**Instability**	**Redislocations**	**Ext. rot. deficit**
			**≤ 5%**	**5-25%**	**≥ 25%**	**Excellent**	**Good**	**Moderate**	**Poor**			
All	25	50	12	10	3	14	7	4	0	4 (16%)	1 (4%)	9°
Operated	13	54	0	10	3	7	4	2	0	1 (8%)	0	6°
Conservative	12	46	12	0	0	7	3	2	0	3 (25%)	1	14°

Ext. rot. deficit: Deficit of external rotation.

Postoperatively, the shoulders were maintained in an orthopaedic sling for six weeks. Within this timeframe, physiotherapeutic assisted abduction was limited to 90° and no external rotation was allowed. After six weeks, rehabilitation was advanced to assisted and then to active exercises without limitations. Non-contact sport was permitted twelve weeks after surgery, contact sport after six months.

The remaining twelve patients with a fracture size smaller than 5% were treated conservatively (Figure 5). All of the patients had a centered glenohumeral joint articulation. The none-operative management included immobilization of the shoulder in an orthopedic sling for six weeks. Assisted passive mobilization avoiding external rotation was started two weeks after trauma. Active assisted and passive external rotation was started after six weeks. Strengthening exercises were routinely initiated after eight weeks, non-contact sports was permitted twelve weeks after trauma. Our therapy algorithm was applied to all patients of our study population. Clinical follow up examination was performed on average 30 months after trauma (range 24–38 months) using the original Rowe score [4] to evaluate the clinical outcome.

Ethical approval was not necessary due to the retrospective study design. All patients signed an informed consent for participating in this study and for the publication of anonymous individual details.

### Statistics

All data underwent statistical analysis using standardized SPSS software 17.0 (SPSS®, Inc., Chicago, USA). Statistical analysis was made using the Mann–Whitney U-test with level of significance P < 0.05 and Kendall’s tau correlation coefficient (R) for comparison of different parameters within each group of patients.

## Results

Twelve patients were treated conservatively (Table 2). They had an average fracture size of 2% (range 1% - 3%) and an average age of 46 years (range 22 – 63 years). After a minimum follow up of two years, average 30 months after trauma (range 24–38 months), seven patients (58%) were classified as having excellent outcomes, three patients (25%) showed good results, and two (17%) had moderate outcomes according to the Rowe score. The average score was 86 (range 51 – 100). The external rotation deficit averaged 14° (range 0° - 60°) in comparison to the healthy other shoulder. Three patients (25%) complained about a persisting feeling of instability. One patient had recurrent dislocations. The plain radiographs showed no evidence of relevant osseous resorption or non-union.

13 patients were treated operatively. The average Rowe score was 89 (range 63–100) after a two years follow up. The results were rated excellent in seven (54%), good in four (31%), and moderate in two cases (15%). The mean external rotation deficit was 6° (range 0° - 50°) in comparison to the healthy other shoulder. One patient complained of a persisting feeling of instability. No recurrent dislocations took place in this group. In two cases (15%) an arthroscopic release was performed due to severe external rotation deficit after three and six months respectively (Table 1). Both patients presented a pronounced external rotation deficit of 30° and 50° and a Rowe score of 85 and 63 after performing surgical release. The fracture size in the operatively treated group averaged 15% (range 8% - 25%). The average age was 54 years (range 31 – 69). Three patients (23%) had a fracture size of 25% or more. At follow-up, they had an excellent average Rowe score of 95 (range 88 – 100) and had no external rotation deficit. Four patients were treated arthroscopically, three patients arthroscopically assisted, and four patients were treated by an exclusively open approach, excluding the two patients who were treated by coracoid transfer. Comparison of these different operative therapy techniques shows similar average Rowe scores (91 arthroscopically, 89 with an arthroscopic assisted approach, and 94 with an open approach) and the external rotational deficits showed no significant differences. There was also no significant difference regarding the defect sizes. The four arthroscopically treated patients had no intra- or postoperative complications. Two other patients had relevant outer rotation deficits. One of them had been treated by an open approach and internal fixation, the other one had a coracoid transfer (Table 1). One patient in the group treated by arthroscopically assisted reduction and osteosynthesis complained of a persistent feeling of instability without recurrent luxation. No statistically significant differences could be evaluated between the patients subjected to an operative versus conservative treatment strategy with respect to complication rate (p = 0.91), Rowe score (p = 0.98), or external rotational deficit (p = 0.17). However, the patients treated by an operative strategy were significant older than the patient group treated conservatively (p = 0.04). There were no statistical differences between patients treated with open, arthroscopically assisted, and exclusively arthroscopical procedures. By evaluating synergies between pre-treatment conditions, such as age and defect size, and clinical outcome, no significant correlation could be seen. A statistically significant correlation was found between the patients’ age and the posttraumatic defect size (p = 0.02).

## Discussion

The vast majority of our operatively treated patients showed an excellent (seven) or good (four) Rowe score after a minimum of twenty-four months. The number of moderate (two) results was low and no cases of inferior results, recurrent dislocations, or infections were seen. The mean Rowe score of our operatively treated patient group was 89 with a mean external rotation deficit of 6°. Similarly, the patients, treated conservatively, offered promising results. The average Rowe score in this group was 86. We have found no statistically significant differences between the patients subjected to a conservative versus operative treatment strategy with respect to clinical outcome and complication rate. However, the external rotational deficit was slightly lower in our operatively treated study group.

In order to offer the patients with osseous Bankart lesion after first-time traumatic shoulder dislocation a consistent and evidence based treatment plan, the authors performed a literature review and created the above mentioned treatment algorithm. There exists some evidence for superior results of osseous Bankart repair in acute cases compared to chronic ones [13] as well as high chance of partial absorption of the bony fragment during the first year after fracture [12]. On the other side it could be shown, that osseous Bankart lesions are associated with a high rate of osseous healing and good clinical outcomes after conservative treatment [9,10]. As a result of limited evidence regarding the effect of the defect size of acute bony Bankart lesion on outcomes, the authors created this algorithm based on the experience on shoulder instability with chronic glenoid rim defects. Sugaya et al. recommended reduction and internal fixation of small or medium sized lesions, as well as grafting procedures in large defects [14]. In order to minimize chronic instabilities and to avoid fracture absorption as well as to take advantage of the high healing potential of acute osseous Bankart lesions after conservative therapy, we recommended a conservative treatment strategy in active patients small osseous bankart lesions (<5%), whereas patients with medium-sized or large bankart fractures were suggested a surgical treatment strategy.

Various surgical techniques for the treatment of anterior glenoid rim fractures have been described in literature. The recommendations range from arthroscopic procedures using anchors, open Bankart repair using anchors or screws, and autologous bone grafting to coracoid transfer procedures [1,14-21]. Itoi et al. [3] have shown in a cadaver model that an optimal reduction of the fracture and capsular-labral complex is essential to reduce the postoperative loss of external rotation. A glenoid defect of 1 cm will lead to a loss of external rotation of 25°.

Generally, studies dealing exclusively acute osseous Bankart lesions are rare. Porcellini et al. [1] treated 25 patients arthroscopically with an average age of 26 years. Inclusion criteria were patients who sustained an anterior shoulder dislocation with associated glenoid rim fracture treated within three months and a fracture size involving less than 25% of the glenoid surface. After an average follow up of two years, 92% of the patients returned to sports at the same level as preoperatively. The mean external rotation deficit was 10°. These results are comparable to ours, regarding both conservativly and surgically treated patients, although our patient population was considerably older. Salomonsson et al. [10] reported outcomes of patients with first-time shoulder dislocation and mostly conservative treatment. This study showed promising outcomes in those patients without recurrent instability. Only a minority of three patients with acute osseous Bankart lesion developed recurrent instability. Unfortunately, no information regarding the size of the bony Bankart fragment was presented. Scheibel et al. [22] included 17 acute and eight chronic osseous glenoid rim fractures in their study. Patients with osseous Bankart lesions of less than 25% of the glenoid surface were treated by an open procedure using anchors while lesions above 25% were refixed with screws. After twenty-two months, patients with a defect size of less than 25% had an average Rowe score of 94 and a mean external rotation deficit of 6°, whereas patients with a defect size of 25% or more had an average Rowe score of 90 and a mean external rotation deficit of 12°.

There are currently no studies dealing particularly with acute Bigliani type IIIb glenoid rim fractures involving 25% of the glenoid surface. However, there exists some outcome data for patients with chronic glenoid rim defects. Rockwood and Matsen [23] stated that fractures involving 25% or more of the glenoid surface should be treated with open reduction and internal fixation. Furthermore, Burkhart and De Beer [15] recommended a Latarjet procedure in cases of a chronic anterior instability and a defect size exceeding 25%. Moreover, Warner et al. [24] treated eleven patients with a glenoid defect size over 25% and chronic anterior-inferior instability with autogenous tricortical iliac crest bone grafts. The average Rowe score after 33 months was 94 and the external rotation deficit averaged 14°. In comparison to these studies which only included chronic cases, our three patients with a defect size involving 25% showed excellent outcomes with an average Rowe score of 95 and an external rotation deficit of 5°.

In conclusion, our results are in accordance with the results of Porcellini, Salomonsson, and studies including chronic osseous Bankart lesions [1,10,15,22,24]. Thus, based on the experiences of chronic glenoid rim repairs, our treatment algorithm for acute osseous Bankart lesions leads to promising clinical results.

The limitations of this study include the limited number of patients included and the retrospective study design. Furthermore, the position of the bony bankart fragment after reduction was not part of our algorithm. Particularly, Maquieira et al. reported of good clinical results of even large osseous bankart leisons treated conservatively if the glenohumeral joint was reduced concentrically. Thus, a higher percentage of patients might benefit from a conservative approach. The strengths of this study are our strict inclusion parameters, such as exclusively acute traumatic glenoid rim fracture caused by first time anterior shoulder dislocations. Additional, a three dimensional computer tomography was performed for all patients, with a glenoid rim fracture on plain radiographs, to evaluate an accurate measure of defect area. Further studies are needed to confirm this treatment strategy in cases of acute glenoid rim fractures.

## Conclusions

Applying our treatment algorithm for acute osseous Bankart lesions consisting of a conservative strategy for small defect sizes and a surgical approach for medium-sized and large defects leads to encouraging mid-term results and a low rate of recurrent instability in active patients.

## Abbreviations

CT: Computer tomography; Ext. rot. deficit: Deficit of external rotation.

## Competing interests

The authors declare that they have no competing interest.

## Authors’ contributions

US participated in the study design, worked through the medical records, identified the patient collective, contacted all patients at the follow-up, performed the statistical analysis, and drafted the manuscript. CR performed a considerable amount of the operative procedures and helped in setting up the treatment strategy. PH participated in performing the statistical analysis, and helped to draft the manuscript considerably. PR conceived of the study, and participated in its design and coordination, performed a considerable amount of the operative procedures and helped to draft the manuscript. All authors read and approved the final manuscript.

## Pre-publication history

The pre-publication history for this paper can be accessed here:

http://www.biomedcentral.com/1471-2474/14/305/prepub
